# Theoretical framework for determining hospital length of stay (LOS)

**DOI:** 10.1186/1753-6561-6-S4-P32

**Published:** 2012-07-09

**Authors:** E Schorr

**Affiliations:** 1 University of Calgary, Canada

## Introduction

Previous literature indicates many factors contribute to inpatient hospital length of stay (LOS). Unfortunately no comprehensive or theoretical model, which highlights the underlying processes affecting these relationships, exists. The purpose of this study was to generate a theoretical framework, using two prevalent medical conditions (acute myocardial infarction (AMI) and cesarean (‘c’) section), of the determinants of LOS.

## Methods

A literature review was conducted using MEDLINE (OVID) and EMBASE databases. Search terms included ‘length of stay’ combined with ‘determining or determinant or indictor. Additionally we used the same key terms as listed above and combined them with either ‘acute myocardial infarction’ or ‘cesarean section’. A grey literature review was also completed. Articles whose primary focuses were not the determinants of LOS were excluded from the study. Articles containing only emergency department and trauma unit data were also excluded.

## Results

Findings from the literature review propose a general model based on four categories of determinants 1) patient characteristics: medical history, healthcare knowledge, family support, religion, age/sex/gender/race, residence, type of procedure and severity of disease; 2) clinical caregiver characteristics: culture, specialty, training, team, quality of care and physician choice of prescribed medications; 3) characteristics of the social or family environment: school, peers, political systems, economics and community and 4) characteristics or properties of the healthcare system: admitting service (surgical vs. medical), structure of services, types of services and available technology, palliative care, insurance, long term care, access, setting occupancy, transfers between hospital and care settings venue (emergency vs. non emergency department) and geographic location (urban vs. rural).

## Conclusions

The literature suggests that LOS is complex in nature, and cannot be determined by a single factor. A model of the determinants of length of stay, which can be applied across various medical settings, will help determine healthcare policy, cost, and planning and will ultimately allow future healthcare services to be both streamlined and standardized.

